# Structural Modifications Introduced by NS2B Cofactor Binding to the NS3 Protease of the Kyasanur Forest Disease Virus

**DOI:** 10.3390/ijms241310907

**Published:** 2023-06-30

**Authors:** Shivananda Kandagalla, Bhimanagoud Kumbar, Jurica Novak

**Affiliations:** 1 Laboratory of Computational Modeling of Drugs, Higher Medical & Biological School, South Ural State University, 454080 Chelyabinsk, Russia; 2 ICAR-National Institute of Veterinary Epidemiology and Disease Informatics, Bengaluru 560064, Karnataka, India; 3 Department of Biotechnology, University of Rijeka, 51000 Rijeka, Croatia; 4 Center for Artificial Intelligence and Cybersecurity, University of Rijeka, 51000 Rijeka, Croatia

**Keywords:** NS3, NS2B, KFDV, AlphaFold, PCA, MD, clustering, allosteric binding site, conformational change

## Abstract

Kyasanur Forest Disease virus (KFDV), a neglected human pathogenic virus, is a *Flavivirus* that causes severe hemorrhagic fever in humans. KFDV is transmitted to humans by the bite of the hard tick (*Haemaphysalis spinigera*), which acts as a reservoir of KFDV. The recent expansion of the endemic area of KFDV is of concern and requires the development of new preventive measures against KFDV. Currently, there is no antiviral therapy against KFDV, and the existing vaccine has limited efficacy. To develop a new antiviral therapy against KFDV, we focused on the nonstructural proteins NS2B and NS3 of KFDV, which are responsible for serine protease activity. Viral proteases have shown to be suitable therapeutic targets in the development of antiviral drugs against many diseases. However, success has been limited in flaviviruses, mainly because of the important features of the active site, which is flat and highly charged. In this context, the present study focuses on the dynamics of NS2B and NS3 to identify potential allosteric sites in the NS2B/NS3 protease of KDFV. To our knowledge, there are no reports on the dynamics of NS2B and NS3 in KFDV, and the crystal structure of the NS2B/NS3 protease of KFDV has not yet been solved. Overall, we created the structure of the NS2B/NS3 protease of KFDV using AlphaFold and performed molecular dynamics simulations with and without NS2B cofactor to investigate structural rearrangements due to cofactor binding and to identify alternative allosteric sites. The identified allosteric site is promising due to its geometric and physicochemical properties and druggability and can be used for new drug development. The applicability of the proposed allosteric binding sites was verified for the best-hit molecules from the virtual screening and MD simulations.

## 1. Introduction

Kyasanur Forest Disease virus (KFDV) is a tick-borne encephalitis virus that belongs to a group of serocomplex flaviviruses and causes severe hemorrhagic fever in humans. The name of the virus originates from the Kyasanur forest area in Shivamogga district of Karnataka, India, where the virus was first detected in sick and dying monkeys in 1957 [1,2]. Annually, 400–500 new human KFDV cases are reported with a mortality rate of about 3% to 5% [3,4]. The virus is classified as a category C priority pathogen by the United States National Institute of Allergy and Infectious Diseases (NIAID). The KFDV virus is zoonotic and vector-borne, circulating between the vector ticks and reservoir-competent vertebrate hosts. The main vector involved in KFDV transmission is *Haemaphysalis spinigera* and is common in India, Sri Lanka, and Vietnam [5]. In addition, KFDV has been isolated from *Dermacentor* (*Dermacentor auratus*) and other tick species of the *Ixodes* genus [6]. Ticks acquire the virus by biting an infected vertebrate during their various developmental stages and eventually transmit the virus to the host. Transstadial maintenance of the virus has also been reported [7]. The outbreak of KFDV in humans mainly occurs in spring and summer, which corresponds to the maximum activity of the tick vector. Previous studies have clearly shown the important role played by the nymph stage (with its maximum from January to May) of the tick in the transmission of the KFDV virus to humans [8]. This shows the close relationship between disease outbreaks and tick activity [9]. Humans become infected with KFDV primarily via monkeys or other mammals through the bite of infected ticks. To date, there have been no reports of human-to-human transmission of KFDV. Previously, KFDV was endemic in the Indian state of Karnataka but has spread to other states, such as Kerala, Maharashtra, Goa, and Tamil Nadu in recent years. Recently, a single human case of KFDV has been reported in China, the first report of KFDV outside India [10]. This expansion of the endemic area of KFDV is of concern and makes the development of new preventive measures against KFDV even more urgent. KFDV falls into the category of neglected human pathogenic viruses, and a deeper understanding of its pathogenesis, ecology, and epidemiology is needed to develop effective preventive measures.

The genome of KFDV consists of a positive-sense single-stranded RNA genome that is 10,774 nucleotides long and encodes a single polyprotein. The encoded single polyprotein chain is 3416 amino acids (aa) long and is post-translationally cleaved into a total of three structural (capsid C, pre-membrane (prM) or transmembrane protein M, and envelope E protein) and seven non-structural (NS) proteins (NS1, NS2A, NS2B, NS3, NS4A, NS4B, and NS5) [11]. The present study focuses on the non-structural proteins responsible for serine protease activity. The non-structural proteins, such as NS2B and NS3, are an important component of the serine protease protein. The NS2B/NS3 protease in *Flavivirus* belongs to the trypsin serine protease superfamily [12]. NS3 is a large multifunctional protein with serine protease, RNA triphosphatase, nucleoside triphosphatase, and helicase activities [13,14,15]. NS2B, on the other hand, acts as a cofactor for the protease activity of NS3 [16,17]. Together, the NS3 protein and NS2B act as a viral protease and auto-cleaves the polyprotein at dibasic sites in the cytoplasm. The 184 aa long NS3 protein and the approximately 40 aa long hydrophobic core of the NS2B protein are part of the viral protease complex. This viral protease complex cooperates with host proteases to cleave the polyprotein precursor produced by the viral genome. The NS2B/NS3 protease cleaves peptide bonds at multiple sites on the viral polyprotein located in the capsid and between NS2A–NS2B, NS2B–NS3, NS3–NS4A, and NS4B–NS5, resulting in the release of mature viral proteins. In general, the substrate binding site of the NS2B/NS3 protease prefers basic residues (Arg or Lys) at the P1 and P2 positions and a short side chain amino acid at the P1′ position [18,19]. The crystal structure of the NS2B/NS3 protease of KFDV has not yet been resolved while both apo- and inhibitor-bound forms have been determined for other flaviviruses [20,21,22,23]. Viral proteases have emerged as suitable therapeutic targets in the development of antiviral drugs against many diseases, such as AIDS and hepatitis [24,25]. However, the development of inhibitors targeting the NS2B/NS3 protease in *Flavivirus* has limited success, Refs. [26,27,28], possibly due to two important properties of the active site [28,29,30]. First, the active site of the NS2B/NS3 protease is flat, making specific inhibitors highly unlikely. Second, the active pocket is highly charged, and its P1 and P2 positions bind mainly to substrates with basic residues, and this charge requirement reduces the bioavailability of inhibitors in vivo [31]. These reports highlight the need to search for alternative allosteric sites in the NS2B/NS3 protease to identify potential inhibitors.

Experimentally solved crystal structures of the NS2B/NS3 protease are not available for KFDV, and crystal structures of the NS2B/NS3 protease of flaviviruses have been described in previous studies [26,28,29]. The *N*-terminal domain of NS3 has a chymotrypsin-like serine protease domain that cleaves the viral polyprotein precursor in both *cis* and *trans* into individual NS proteins (the cleavage sites are described above). A *C*-terminal NTPase-dependent RNA helicase domain is involved in viral RNA synthesis and genomic replication [30]. The chymotrypsin domain of the flavivirus NS2B/NS3 protease lack the intrinsic capacity to correctly fold without the NS2B cofactors [32,33,34]. NS2B, an integral membrane protein (14 kDa), has three domains, two transmembrane *N*- and *C*-terminal domains, and a central core domain (≈47 amino acid long) that functions as an essential protein cofactor of the NS3 protease [35]. In the substrate binding site of the NS3 protease, the *C*-terminal portion of the central core domain of NS2B forms a β-hairpin that contributes to the formation of the hydrophobic S2 and S3 pockets, demonstrating a direct catalytic role of NS2B [23,27,36]. In the absence of NS2B, the flavivirus NS3 protein does not fold properly and is therefore neither soluble nor catalytically active as a protease in vitro [37]. The flavivirus NS2B/NS3 protease adopt two distinctive conformations: the “opened” and the “closed” states [23,27]. In both conformations, the NS2B cofactors assumes diverse conformations, whereas the NS3 protease adopts the similar chymotrypsin fold. Crystal structures of the NS2B/NS3 protease of flaviviruses were described in apo- and inhibitor-bound forms [26,28,29]. The *C*-terminal part of NS2B attains an “opened” conformation (inactive) in the absence of a substrate or active-site inhibitor. On the other hand, the *C*-terminal portion of NS2B wraps around the NS3 core upon interaction with the inhibitor or substrate, closing the NS3 active site in the so-called active “closed” conformation. Thus, conformational changes induced by NS2B are required for NS3 function. NS2B regulates NS3 protease activity by stabilizing the correct protein conformation and participates in substrate cleavage. The identification of molecules that disrupt the interaction between NS2B cofactors and NS3 chymotrypsin domains by preventing proper folding of the enzymes is a promising strategy for the development of antiviral inhibitors against flavivirus proteases. Allosteric inhibition of viral protease is one such strategy.

In view of the above reports, the present study mainly focuses on the dynamics of NS2B and NS3 to elucidate the influence of the NS2B cofactor on the tertiary structure of NS3 of KFDV and to identify potential allosteric sites in the KDFV NS2B/NS3 protease. To this end, the structure of the NS2B/NS3 protease of KFDV was modeled using AlphaFold [38]. The recent development of AlphaFold, an artificial intelligence system developed by DeepMind, alleviates the problems with existing homology modeling tools. It predicts the three-dimensional structure of proteins based on their amino acid sequences with a high accuracy that rivals experiment, and its predictive power was determined by version 14 of the Critical Assessment of Protein Structure (CASP) experiment. Recently, 98.5% of the proteins in the human proteome were assigned to predict structures using AlphaFold, and the predicted structures can be accessed through a portal hosted by the European Bioinformatics Institute (http://alphafold.ebi.ac.uk (accessed on 18 January 2022)). With the above goal in mind, we constructed the structure of the NS2B/NS3 protease of KFDV using AlphaFold and performed molecular dynamic simulations with and without the NS2B cofactor to identify alternative allosteric sites.

## 2. Results

### 2.1. AlphaFold Predicts a Reliable Model of the NS2B/NS3

The amino acid region considered for modeling (1363 to 1660 aa) comprises the transmembrane portion of NS2B and was excised after obtaining the model. The overall confidence score of the model was reliable and is shown in Figure S1 in the Supplementary Information. The main structural properties of the model are listed in Table 1, which were calculated using the MolProbity v.4.5.2 and PROCHECK v.3.5 servers. The MolProbity analysis shows the most favorable values for 5 of 6 properties, including the overall MolProbity result (Table 1, bold entries). Similarly, the PROCHEK program shows the distributions of the dihedral angles of the model based on the Ramachandran plot. The correct folding of NS2B and NS3 is essential for protease activity and was verified by overlaying the generated NS2B/NS3 protease model with analogous proteins from other *Flavivirus* families. The crystal structure deposited in the Protein Data Bank under ID 6JPW was used for the superimposition because it has a 50% sequence similarity to NS3 from KFDV. The structure overlay clearly shows the correct folding of NS2B and NS3 in the created model and is shown in Figure S2 in the Supplementary Information. Overall, the model generated using AlphaFold appears to be reliable in terms of geometry from a protein structure perspective.

### 2.2. NS2B Cofactor Binding Reduces NS3 Flexibility

**Figure 1:**
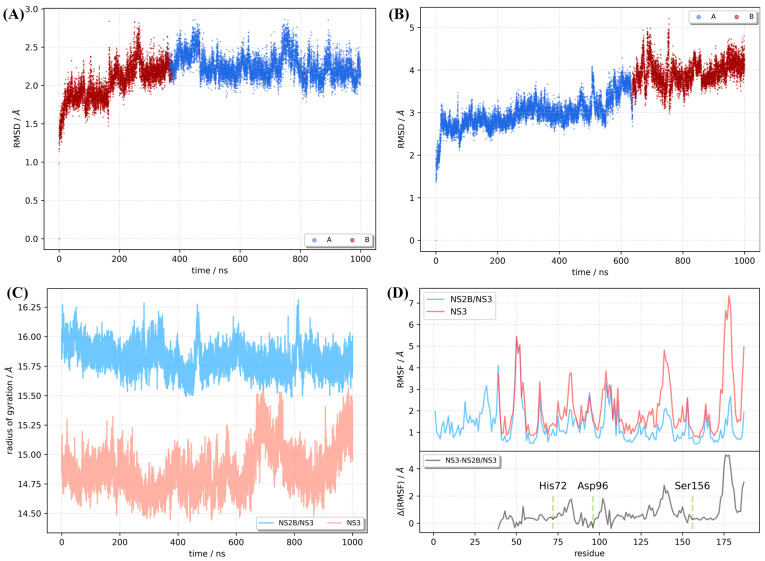
MD trajectory analysis. Backbone RMSD with conformer population along trajectory for KFDV NS3 (**A**) and NS2B/NS3 (**B**) proteins. Radius of gyration (**C**). Root mean square fluctuations per residue ((**D**), upper panel). Difference of RMSF values for KFDV NS3 protein for naked and NS2B-complexed NS3 protein ((**D**), lower panel).

The stability and conformational dynamic properties of the NS3 with and without NS2B were investigated using MD simulation. The evolution of root mean square deviation (RMSD) and radius of gyration (RoG) of backbone atoms of NS3 in free form and with bound NS2B cofactor are shown in Figure 1A,B, respectively, demonstrating the overall structural fluctuations. The RMSD of NS3 with an NS2B cofactor (NS2B/NS3 complex) is quite constant with an average value of 2.17 Å and standard deviation of 0.22 Å. The dynamics of naked NS3 without an NS2B cofactor shows much greater flexibility. This fact is reflected in the increase in the average RMSD values of NS3 from 2.73 ± 0.26 Å (for the first 550 ns of the dynamics) to 3.56 ± 0.48 Å (for the dynamics from *t* = 550 ns to *t* = 1000 ns). The conclusions are additionally confirmed by analysis of radius of gyration, a property describing the compactness of the system. Since NS2B/NS3 complex is more voluminous, it has higher RoG values (Figure 1C). It is important to notice smaller variations in the RoG of NS2B/NS3 along the trajectory, suggesting that NS2B cofactor increases the stability of NS3 compared to naked NS3. In addition, the flexibility of each residue was described by root mean square fluctuation (RMSF), shown in Figure 1D. From the lower panel of Figure 1D, where the difference between the RMSF of naked NS3 and NS2B/NS3 complex (ΔRMSF) is plotted, it is obvious that NS2B cofactor significantly reduced the flexibility of side chains. While the average difference is 0.78 Å, residues from catalytic triad have less than average reduction of flexibility, what could be understood from evolutionary point of view. If we disregard the fluctuations at the *C*-terminus of the protein, our analysis points to two regions with ΔRMSF above 2 Å. These regions include fragments from Gly49 to Arg55 and Leu138 to Gly141, located on the opposite side of the groove, which is the binding site of the *C*-terminal side of the NS2B cofactor.

The binding affinity of the NS2B cofactor was estimated using the well-known MM/GBSA approach. The free energies of solvation are calculated by solving the generalized Born equation and the van der Waals, electrostatic, and polar and nonpolar contributions sum to give the final free energies of binding. Due to the high computational cost, the unfavorable entropy contribution (−TΔS) was neglected. The estimated free energy of binding of the NS2B cofactor to the NS3 protein is −150.0 ± 8.5 kcal mol^−1^.

To better understand the cofactor-enzyme binding process, a decomposition of the MM/GBSA binding free energy was performed. We identify the top 5 residues (Table 2) of both the enzyme (NS3) and cofactor (NS2B) sides that contribute the most to the free energy of binding (ΔG_bind_). Five NS2B residues (Leu2, Ala4, Trp6, Val10, Trp12) have ΔG_bind_ below −4.9 kcal mol^−1^, with ΔG_bind_ of Trp12 being the most negative (−8.7 kcal mol^−1^). In addition to Arg44 of NS3 (ΔG_bind_ = −7.8 kcal mol^−1^), residues Tyr43, Val80, Gln116, and Ile137 have ΔG_bind_ in the range between −4.3 kcal mol^−1^ and −4.6 kcal mol^−1^.

The number of hydrogen bonds was calculated using pytraj [39], a Python package binding to the cpptraj program [40], with default criteria for defining hydrogen bonds. On average, there are 16 hydrogen bonds between the NS2B cofactor and the NS3 protease, with both molecules acting as hydrogen donors in eight cases.

### 2.3. Clustering Identifies the Conformations of NS2B/NS3 and NS3

**Figure 2:**
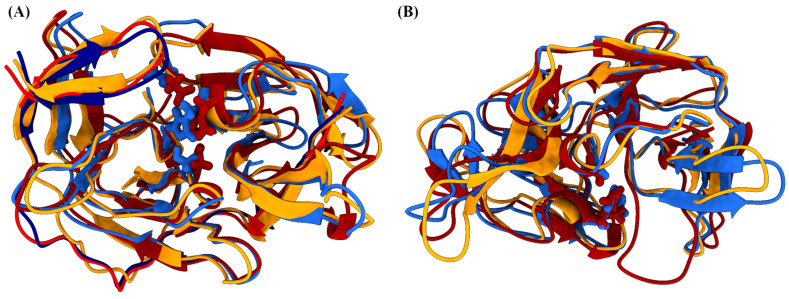
Superposition of the structures of the representative conformations (A—blue; B—red; initial structure—yellow) of the NS2B/NS3 complex extracted from molecular dynamics simulation. (**A**) NS2B/NS3 complex, (**B**) NS3.

The *k*-means clustering analysis of the MD trajectories reveals two conformations for the NS2B/NS3 complex and two for NS3. The number of clusters was determined using the Davies–Bouldin index (DBI), the pseudo-F statistic (pSF), and the ratio of the sum of squares regression and sum of squares error (SSR/SST); the results are summarized in Figure S3 (Supplementary Information). The conformations obtained from the cluster centroids were superimposed on the original conformation for detailed study. First, two different conformations of the NS2B/NS3 complex were considered, and their distribution over the simulation time is shown in Figure 1A. The predominant conformation, A, was populated 63.6% of the simulation time while the other conformation, B, has a population of 36.4%. The structural differences between the conformations inside and outside the binding site are significant (Figure 2). The protease of the *Flavivirus* family virus is a chymotrypsin type serine protease with a classical catalytic triad (Ser-His-Asp), so we find from the representative cluster geometries that the geometry of the catalytic site is conformation dependent. For example, the distance between the oxygen OG of Ser156 and the nitrogen NE2 atom of His72 residue is 2.62 Å while the analogous distance in conformer B is 2.86 Å. The carbonyl oxygen of Asp96 is only 2.85 Å away from the ND1 atom type of His72 in confomation B and 7.89 Å in the dominant A conformation, which is due to the realignment of the Asp96 side chain. The geometric change is directly triggered by the presence of the NS2B cofactor, as Asp96 forms a hydrogen bond (O···HN distance is 2.25 Å) with the NH_2_ group of the Gln29 residue. After the binding of NS2B binding to NS3, the unstructured loop at the *C*-terminus of the NS2B cofactor is located on top of the Asp96 residue. The Asp96 residue is additionally fixed in this orientation because it serves as a hydrogen acceptor for two hydrogen atoms of the Arg141 guanidine group. Five residues preceding Asp96 in the primary structure (Asp91-Glu95), together with the unstructured loop (Ser47-Gln56), contribute the most to the increase in RMSD for two conformations.

From the available crystal structures, it is known that the *C*-terminal part of NS2B can adopt two different conformations in the absence of a substrate or inhibitor (e.g., an open and a closed one) [41]. However, we did not find such conformation of NS2B during simulations (1 μs) because it is believed that conformation change is induced only after substrate binding, in a so-called induced fit mechanism [20].

Subsequently, two conformations of the NS3 without NS2B cofactor were determined with a cluster analysis (Table 3), and their distribution along the simulation is shown in Figure 1B. The dominant NS3 conformation (NS3-A) is characterized by a distance of 2.86 Å between Asp96-O and His72-ND1. This is the only hydrogen bridge formed by the Asp96 residue. Ser156 hydroxy group is 4.35 Å away from the His72-NE2 atom. In the NS3 B conformation (NS3-B), the carboxylic oxygen atoms of Asp96 are 2.79 Å and 3.12 Å away from the His72-ND1 atom. Ser156 is close to the His72-NE2 atom (3.48 Å), and a hydrogen bond could be formed with a small rearrangement. Together with the differences in the relative positions of the catalytic residues, the transformation of the secondary structure of the Gly172-Ser184 fragment from two antiparallel β-sheets connected by turn to an unstructured loop with 11 residues has a tremendous impact on the “openness” of the catalytic triad to the potential substrate. The two lobes of the NS3 protein are connected by a hydrogen bond between the Gly174 oxygen of the peptide bond and the NH_2_ group of the Arg75 side chain.

NS3-A differs from the A conformation of NS3 extracted from the MD simulation of the NS2B/NS3 complex, as the RMSD between the two geometries is 2.43 Å. Again, the unstructured loops showed the greatest variability. Nevertheless, the antiparallel β-sheet motif (Ala78-Gly87) moves closer to the Tyr46 (β-sheet) in NS3-A. This motion is prevented in the NS2B/NS3 form of NS3 because the *N*-terminus of the NS2B cofactor is bound in this groove. The same structural rearrangement is much less pronounced in NS3-B. However, the RMSD of NS3-B compared to the NS2B/NS3-A conformation (3.70 Å) indicates rather large changes. After considering the representative geometries, two antiparallel β-sheets (Gly174-Lys176 and Tyr181-Ser183) became part of a long unstructured loop.

### 2.4. PCA Shows Large-Amplitude Motion Changes after Cofactor Binding

**Figure 3:**
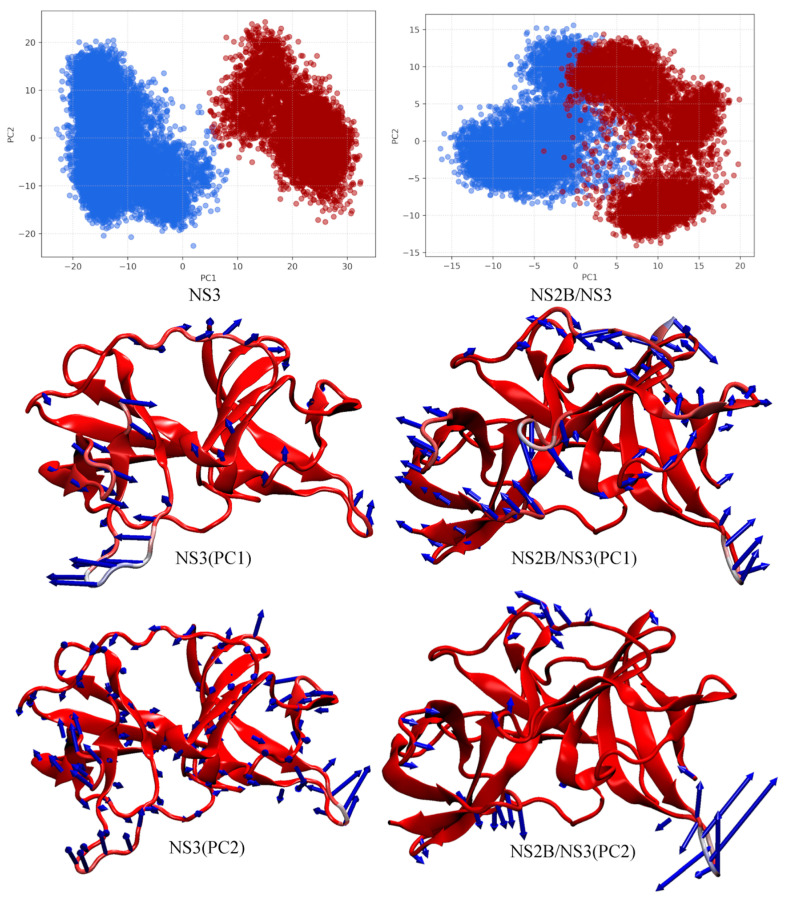
PCA analysis of trajectories from MD simulations. NS3 (**left**) and NS2B/NS3 (**right**). Two-dimensional subspace spawned by the first two principal components PC1 and PC2 (**top**, conformation A—blue; B—red). Visualization of molecular motion associated to the first (**middle**) and the second (**bottom**) PCs. The degree of motion is depicted by the length of the arrow.

From a dynamics point of view, a principal component analysis was used to isolate the large-scale motions of the naked NS3 and NS2B/NS3 complexes. Initially, up to ten principal components (PC) were calculated, but only the first two components could characterize the large-scale motions of the proteins. For NS3, the first two principal components explained 44.4% and 9.6% of the total variance, respectively. For NS2B/NS3, the relative ratio of principal components changed, with PC1 explaining 21.1% and PC2 14.0%. The projections of PC1 and PC2 for both systems from MD simulations into two-dimensional subspace plots are shown in Figure 3. In addition, color coding with conformational information from *k*-means clustering analysis is added. A simple summation of the percentage of variance explained by PCA1 and PCA2 proves that the two-component approximation is better for the naked NS3 protein with 54% explained variance. Two identified motions of an NS3 protein are leading motions for conformational changes. Coordinate projections for CA atom types were obtained using the cpptraj script and visualized using the ‘nmwiz’ plugin for VMD [42]. Consistent with previous observations of differences and similarities between conformations, principal components isolated large amplitude fragment motions near the groove to which the NS2B cofactor binds.

### 2.5. Potential Druggable Pockets

**Figure 4:**
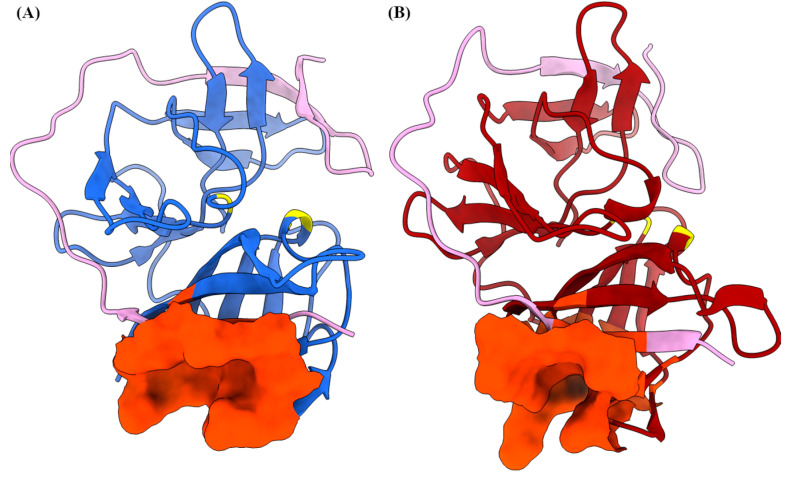
Representative conformations with substrate binding site (or catalytic triad) highlighted in yellow. The representative conformations (A—blue; B—red) of the NS2B/NS3 complex extracted from molecular dynamics simulation (**A**). The NS2B cofactor is highlighted in light pink and predicted BP1 allosteric site highlighted in orange red (depicted in surface view) (**B**).

All conformations obtained from the *k*-means cluster analysis were used to search alternative druggable pockets, as these are the intermediate conformations that can be observed during the activation of NS2B/NS3. We excluded the obtained conformations from the MD simulation of NS3 without NS2B complex for the analysis because the flexible NS2B cofactor is essential for the activity of NS3 protease without which NS3 protease cannot fold properly to the functional active chymotrypsin-like conformation [43]. First, potential binding pockets of two conformations of the NS2B/NS3 complex were identified using the DoGSiteScorer program (https://proteins.plus (accessed on 3 August 2023)). The DoGSiteScorer program identifies the potential binding pockets/subpockets of a protein. It then analyzes the geometric and physicochemical properties of the binding pockets and estimates their druggability [44]. The predicted binding pockets located within the active site were not considered in the analysis. The identified binding pockets from two conformations of the NS2B/NS3 complex are listed in the Table 4. Additional geometric and physicochemical properties of the binding pockets are provided in the Supplementary File S1 (Tables S3 and S4). We found that some binding pockets are conserved between the two different conformations of the NS2B/NS3 complex, such as the binding pocket (BP1) of conformation A with the BP1 of conformation B, similarly the BP2 of confirmation A with BP5 of confirmation B. Based on the drug score, the pockets, such as a BP1 of conformation A and BP1 of conformation B, can be used as an alternative allosteric pocket for targeting the NS2B/NS3 complex. The location and amino acids in the BP1 of conformation A and the BP1 of conformation B are almost identical, but some amino acids are part of this binding pocket only in particular conformations due to structural changes during the simulation (Figure 4). Additionally, some amino acids that play a crucial role in NS2B/NS3 binding based on MM/GBSA free energy analysis (Table 2) are part of the BP1 binding pocket in conformation A and B (highlighted in Table 4). Interestingly, a similar pocket (NS2B 2B51 and 2B53 pocket) has been identified on the dengue-3 and Zika NS2B/NS3 protease to bind emoporfin, niclosamide, nitazoxanide, erythrosin B, and JMX0207 from the Schrödinger Maestro SiteMap panel [45,46,47]. Hence, targeting the BP1 binding pocket could disrupt the necessary interaction between NS2B with NS3. Overall, the identified BP1 binding pocket is considered promising due to its geometric and physicochemical properties and druggability and can be used for the development of new drugs.

### 2.6. Allosteric Binding Sites—Hit Molecules Identification

**Figure 5:**
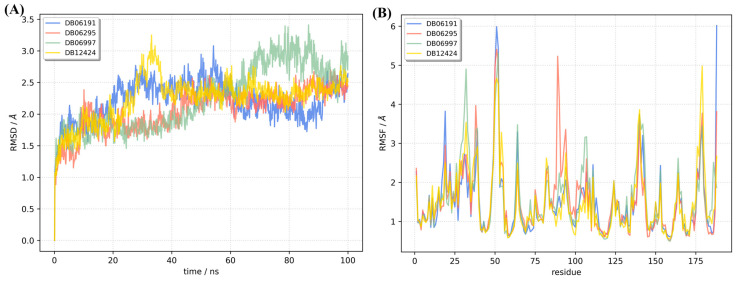
Analysis of the MD trajectories of conformations A of the NS2B/NS3 protease with selected potential allosteric inhibitors. RMSD (**A**) and RMSF (**B**).

**Figure 6:**
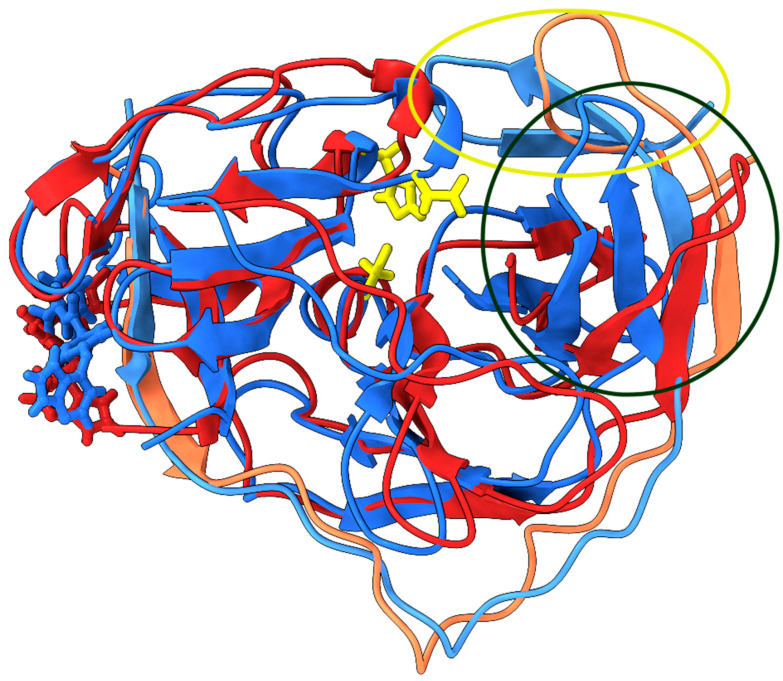
Comparison of structures from the initial (blue) and the last conformations (red) from 100 ns molecular dynamics simulations run for the NS2B/NS3 protease with potential allosteric inhibitor (DB06997), with encircled areas (yellow and black circles) with the greatest structural rearrangements.

The DrugBank database contains compounds that are already approved as drugs or agents that are in various phases of clinical trials. Therefore, their ADME properties and cytotoxicity are well studied. The approach taken in this work to search for new allosteric NS2B/NS3 inhibitors is an example of a drug repurposing study [48,49,50,51] that seeks new potential applications for existing drugs. The first step in our framework was molecular docking. More than 9000 compounds were docked to the allosteric binding pocket of conformation A of the NS2B/NS3 protease of KFDV. The mean binding energy of the potential allosteric inhibitors is −5.7 ± 0.8 kcal mol^−1^. The distribution of binding energies is shown in Figure S4 and as a csv file in Supporting Information. One of the drawbacks of the docking protocol was that it was performed with a fixed geometry of the target receptor. This fact and other known docking issues [52,53] prompted us to verify the docking results with molecular dynamics simulations. The top hit molecules were also docked to the allosteric pocket of conformation B of the NS2B/NS3 protease. The best 4 compounds (DB06191, DB06295, DB06997, DB12424; Table 5) with the most favorable binding energies were selected and 100 ns long MD simulations were performed for complexes of NS2B/NS3 and hit molecules. The RMSD and radius of gyration were monitored throughout the trajectory. All complexes are stable. Only small deviations when compared to the initial structure (Figure 5 and Supplementary Figure S5) support those findings. RMSDs are below 3.5 Å for all complexes, and the RMSD for the NS2B/NS3:DB06295 complex never exceeds 2.8 Å. A slight increase in the radius of gyration is observed for the NS2B/NS3:DB06997 complex. A comparison of the first and last geometries of the trajectory shows that the binding of DB06997 to BP1 affects the structure and dynamics of the NS2B/NS3 protease. Although only the *N*-terminus residues of the NS2B cofactor are in direct contact with the potential allosteric inhibitor, the *C*-terminus undergoes the greatest structural rearrangement (Figure 6). The last two antiparallel β-sheets have lost their secondary structure and moved away from residues His72 and Asp96, making the catalytic pocket more accessible (yellow oval on Figure 6). Even more intriguing is the large amplitude motion of the loop connecting two antiparallel β-sheets of the NS3 protease and shielding the Asp96. The distance between the Asn140 Cα-atom of the loop and the Cα-atom of Arg94 is 8.37 Å in the initial geometry. At only 100 ns after the binding of DB06997 to BP1, the Asn140 loop moved away, making the catalytic pocket accessible to the solvent, and the distance between the two atoms increased to 20.07 Å (dark green oval on Figure 6). Analogous motions were observed in other complexes but on a much smaller scale. The analysis of the RMSF additionally supports previous findings. The RMSF measures atomic positional fluctuations, and mass-weighted fluctuations have been averaged by residue. We refer to residues with an RMSF higher than 3.2 Å as very flexible since the averaged RMSF values are within the 1.49 Å to 1.57 Å range. Residues Ala31-Met32-Gly33 of the NS2B cofactor in NS2B/NS3:DB06997 complex are very flexible, and the RMSF value of Met32 reaches 4.9 Å. The effect is less pronounced in NS2B/NS3:DB12424, where only Gly33 was classified as very flexible. The high flexibility of two loops (Leu50-Leu51-Trp52 and Asn140-Gly141-Arg142) of NS3 is common to all four complexes. The Asn140-Gly141-Arg142 loop moves away from Arg94 exposing the catalytic pocket to the solvent. The Leu50-Leu51-Trp52 loop connects two antiparallel β-sheets in proximity of the allosteric binding site.

The binding affinity of the hit molecules was estimated using the MM/GBSA approach. For the complex NS2B/NS3:DB06295, the free energy of binding without entropy contribution was −26.3 kcal mol^−1^. DB06191 has the highest free energy of binding of all the compounds studied (−15.5 kcal mol^−1^) while the free energies of binding of DB06295 and DB12424 are similar (−24.2 kcal mol^−1^ and −24.6 kcal mol^−1^, respectively). The Van der Waals contribution to the total free energy is found to be more significant than the electrostatic contribution except for DB06997 (Table S1, Figure S6). The non-electrostatic solvation energies for all four hit compounds are comparable. Notably, DB06295 exhibits the highest van der Waals contribution and lower-than-average electrostatic contributions. The MM/GBSA binding free energy decomposition was used to identify key residues with dominant contribution to protein-ligand binding. Table 6 lists the residues with dominant contributions for DB06191, DB06295, DB06997, DB12424. In addition to the polar and uncharged Thr3 and hydrophobic Trp6, five hydrophobic residues (Ala4, Trp6, Tyr43, Tyr46, Val81) and positively charged His54 and negatively charged Asp40 and Glu5 have atoms within 5 Å of DB06997. This finding confirmed the results of previous analyses on the importance of van der Waals interactions. A complete list of the residues’ contribution to the free energy of the binding of hit molecules is provided in the Supplementary Files S2–S5.

## 3. Discussion

The development of inhibitors targeting the NS2B/NS3 protease in flaviviruses has faced limited success due to two significant properties of the active site, as discussed in the introduction. Therefore, this study aimed to address the challenges associated with the active site by focusing on the development of novel allosteric inhibitors, leveraging the structural dynamics of the NS2B/NS3 protease of KFDV.

Several studies in the past have identified potential allosteric inhibitors against the NS2B/NS3 protease of flaviviruses. Roy et al. reported six noncompetitive allosteric inhibitors (myricetin, quercetin, luteolin, isorhamnetin, apigenin, and curcumin) against the Zika NS2B/NS3 protease [54]. All six inhibitors bind to the pockets on the back of the active site of the Zika NS2B/NS3 protease complex. More specifically, based on the results of docking studies, they identified residues such as Leu74-Leu78 and Asp83-Leu86 of NS2B, which form a short β-sheet (near the active pocket), in direct contact with active site inhibitors (cn-716) on one side and with allosteric inhibitors on another side. A similar allosteric binding pocket has been reported for the dengue NS2B/NS3 protease based on docking studies [55,56]. Similarly, Brecher et al. identified the allosteric binding site on the NS3 surface opposite to the active side by comparing the crystal structures of the ligand-bound (PDB: 3U1I) and ligand-free (PDB: 2FOM) forms of dengue protease complexes [57]. However, in all of the above studies, the binding interactions between the allosteric inhibitors, and the NS2B/NS3 protease were not validated by MD simulations after docking. Recently, Millies et al. identified the potential proline-based allosteric inhibitors based on docking and MD simulations, and the allosteric binding site was located on the back of the active site of dengue and Zika NS2B/NS3 proteases [58]. Similarly, several noncompetitive inhibitors and allosteric binding sites have been reported against NS2B/NS3 proteases of flaviviruses. The full list is provided in Table S2 in Supplementary Information.

To our knowledge, the NS2B/NS3 protease of KFDV has not yet been published. This study contributed with a highly reliable 3D structure of the KFDV NS2B/NS3 protease, which was validated by a structural comparison with the genetically related Zika virus NS2B/NS3 protease and by a 1000-ns MD simulation under physiological conditions. The analysis of RMSD, RoG, and RMSF resulted with two crucial findings—the dynamics of the naked NS3 protease is highly influenced after cofactor NS2B binding in a way that flexibility of NS3 residues is reduced. At the same time, the flexibility of the catalytic residues is barely changed, indicating that activation of NS3 protease by NS2B cofactor must include other mechanisms than alternating the geometry of the catalytic triad. A step forward was taken by proposing potential allosteric binding pockets corresponding to those already identified in dengue and Zika virus NS2B/NS3 proteases. BP1, the most promising pocket identified with favorable druggability and geometric and physicochemical properties, is located in the *N*-terminus of the NS2B cofactor, on the opposite side of the protease catalytic pocket. It is very similar to the binding pockets identified on the dengue and Zika NS2B/NS3 protease for which the binding of emoporfin, niclosamide, erythrosin B, JMX0207, and nitazoxanide has been demonstrated [45,46,47].

The feasibility of the suggested allosteric binding sites was validated through virtual screening of DrugBank molecules. The DrugBank database contains compounds that are already approved as drugs or agents that are in various phases of clinical trials. Therefore, their ADME properties and cytotoxicity are well studied. The approach taken in this work to search for new allosteric NS2B/NS3 inhibitors is an example of a drug repurposing study [48,49,59] that seeks new potential applications for existing drugs. The first step in our framework was molecular docking. More than 9000 compounds were docked to the allosteric binding pocket of the NS2B/NS3 protease of KFDV. The top four compounds (DB06191, DB06295, DB06997, DB12424; Table 5) exhibiting the most favorable binding energies were chosen, and the subsequent 100 ns-long molecular dynamic (MD) simulations were conducted for the complexes of NS2B/NS3 with these hit molecules. The free energy of binding for DB06295, DB06997, and DB12424 was estimated to be below −24 kcal mol^−1^, without considering the entropic contribution. DB06997 is recommended for experimental confirmation of the inhibition potential of the KFDV NS2B/NS3 protease because the short 100-ns simulation MD indicates substantial conformational changes of the protease and the geometry of the catalytic pocket triggered by the binding of DB06697 to the allosteric binding pocket.

In conclusion, we would like to point out three important facts. First, docking studies need to be verified by more reliable methods. Recently, Macip et al. [53] demonstrated that docking scores do not correlate with the free energy of binding for the SARS-CoV-2 main protease inhibitors. In our case, the top four compounds with the highest docking score to conformation A of NS2B/NS3 protease are within 0.5 kcal mol^−1^, but their estimated free energies of binding differ by 10 kcal mol^−1^. Ensemble docking further spreads the docking scores, but the calculation of MM/GBSA free energy reordered the potential allosteric inhibitor of NS2B/NS3. Second, MD simulations are essential to see how binding affects the dynamics of the target protein. For DB06997, significant changes are observed on a sub-100 ns time scale. For other simulated complexes, the structural effects are less pronounced. This does not necessarily mean that these compounds are poor allosteric inhibitors, but the 100-ns simulations may be too short to capture conformational changes. Third, for allosterically bound small molecules, it is impossible to tell whether they are inhibitors or agonists based on MD simulations alone. It is known from the literature that NS3 protease activity is regulated by conformational changes. DB06997 induced a structural rearrangement of the complex, but only in vitro experiments can provide a definitive answer as to whether DB06997 binding is favorable for KFDV or whether it inhibits (ideally completely stops) the maturation of the virion.

## 4. Materials and Methods

### 4.1. Prediction of the Structure of the NS2B/NS3 Protease with AlphaFold

The annotated protein sequence of the NS2B/NS3 protease of KFDV was taken from the NCBI Gene Bank (accession ID AFF18434) [11]. The amino acid region in the polyprotein region from 1363 to 1660 was selected for structure prediction using AlphaFold [38]. Both *N*- and *C*-termini of NS2B, which are transmembrane domains in nature, were removed, and only the cytoplasmic loop, which has the role of cofactor, was retained. Structure prediction was performed using the AlphaFold source code in the Google Collab notebook available through a portal hosted by the European Bioinformatics Institute (http://alphafold.ebi.ac.uk, accessed on 17 September 2021) [59]. AlphaFold does not consider a template for model prediction and refines the model with the Amber-relax option to improve the accuracy of side chains geometry. AlphaFold reports the confidence score of each residue based on the pLDDT (predicted local-distance difference test). The pLDDT score ranges from 1 to 100, with 1 representing low confidence and 100 representing high confidence. The quality of the models was additionally assessed using the PROCHECK [60] and MolProbity [61] servers.

### 4.2. Molecular Dynamics (MD) Simulation

MD simulations of the model NS3 protease with and without NS2B cofactor were performed using the AMBER16 program [62]. Simulations were performed according to the slightly modified protocol from [63], in which only the NS3 protease was simulated without the NS2B cofactor. The main change in the current simulation protocol is the longer duration of the productive simulation (1000 ns) while all other parameters, such as periodic boundary conditions, NVT conditions, pressure, and temperature remained the same. The AMBER ff14SB force field [64] was used to parameterize the system. Prior to the simulations, the protonation state of the NS2B/NS3 protease was determined to normal physiological pH using the APBS-PDB2PQR software suite (https://server.poissonboltzmann.org/pdb2pqr (accessed on 15 August 2022)) [65]. The trajectories were processed and analyzed using the CPPTRAJ module from Amber Tools [40].

### 4.3. Principal Component Analysis (PCA)

PCA was performed for the MD trajectories using the pytraj and CPPTRAJ modules [40]. Before analysis, all structures of NS3 protease and NS2B were extracted from MD trajectories and superimposed on the reference structure (first structure from the MD simulation) to eliminate all translations and rotations of the Cα atoms. Only then was the covariance matrix calculated. The atomic fluctuations of the Cα atoms of the individual residues were used to create a covariance matrix. The diagonalization of the covariance matrix resulted in a set of orthogonal eigenvectors (principal components) with corresponding eigenvalues.

### 4.4. Clustering Analysis

The k-means cluster analysis was performed using pytraj and the CPPTRAJ module for each MD trajectory to determine the number of conformations and their relative populations from the trajectories. The optimal number of clusters was determined based on metrics, such as DBI, pSF, and SSR/SST [66]. The maximum number of iterations was set to 1000, with the random randomized initial set of points and sieving set equal to 10. Frames closest to the cluster centroids were identified and considered as representative structures for conformations.

### 4.5. Identification of Potential Druggable Pockets

Binding pockets were identified for the structures determined with *k*-means clustering using the DoGSiteScorer program [44] using the default settings available on the Proteins plus server (https://proteins.plus/ (accessed on 3 August 2022)).

### 4.6. Allosteric Binding Sites—Molecular Docking and MD Simulation

Molecular docking studies identified potential compounds targeting the identified allosteric binding pockets of the NS2B/NS3 complex. First, a drug database was compiled from DrugBank (release version 5.1.9, https://www.drugbank.ca/ (accessed on 15 August 2022), which contained information on 11,913 potential drugs. The collected drug molecules were screened for redundancy and a total of 9407 drugs were selected for molecular docking studies. SMILES were converted to a 3D structure using RDkit, followed by the generation of five conformations and subsequent optimization using the MMFF94 force field. The lowest energy conformations were saved in pdb format. All compounds were prepared for non-covalent docking using the AutoDockTools 4 program’s script prepare_ligand4.py [67] and saved in the pdbqt file format. Similarly, the dominant conformation (A) of the NS2B/NS3 complex identified based on *k*-means clustering during a course of 1 µs MD simulations was considered for docking. The NS2B/NS3 receptor was prepared for docking by adding the Gasteiger charges to all atoms, followed by merging the nonpolar hydrogens. All docking experiments were performed using AutoDock Vina [68] on the University of Rijeka ‘Bura’ supercomputer, based on Intel Xeon E5-2690v3 processors running at 2.6 GHz. A grid box of size 20 × 20 × 20 Å^3^ was defined with the center of the box positioned on the OD2 atom type of the Asp82 residue (x = 48.9, y = 20.6, z = 48.2). It contains all amino acids identified in the allosteric binding pocket (BP1). The grid spacing was set to 1 Å, and the number of modes and exhaustiveness were both set to 20.

The top-ranked compounds were also subjected to docking with conformation B of the NS2B/NS3 complex. The total docking score was calculated using the following formula:

$$score=p_{A}\times {score}_{A}+p_{B}\times {score}_{B}$$

where p_A_ and p_B_ represent the populations of conformations A and B, respectively, and score_A_ and score_B_ correspond to the docking scores for binding in the allosteric pocket of conformation A and B of the NS2B/NS3 protease, respectively.

In addition, the drugs that exhibited the highest binding energy against the allosteric binding pocket of the NS2B/NS3 complex were subjected to 100 ns MD simulations. The simulation parameters, such as parameterization of the protein, periodic boundary conditions, NVT equilibration, pressure, and temperature, were same as described above in “Molecular Dynamics (MD) Simulation” section. Top 4 hit molecules were parameterized with GAFF force field [69]. The binding energy (ΔG_bind_) of the simulated drug and NS2B/NS3 complexes was calculated using the MM/GBSA protocol [70]. A detailed description and methodology of the binding energy calculations can be found in our previous work [63].

## 5. Conclusions

The present study is the first report of the dynamics of NS2B and NS3 in KFDV, as far as we know. Overall, the molecular dynamic simulations provide a comprehensive overview of the structural dynamics of the NS2B/NS3 protease and reveal conformational changes of NS3 with and without the NS2B cofactor. Each of the analyses we performed, such as the RMSD, RoG, RMSF, and PCA of MD trajectories, reveal a piece of the puzzle about the influence of NS2B on the dynamics of the NS3. Moreover, it identified crucial amino acid residues that significantly contribute to the binding of NS2B/NS3, emphasizing the need for further mutational studies to gain a deeper understanding of the binding interactions involving the NS2B/NS3 protease. The *k*-means cluster analysis suggests two dominant conformations for the NS2B/NS3 complex and NS3. In addition, the investigation of potential binding pockets in two different conformations of the NS2B/NS3 complex revealed the binding pocket that is conserved between the different conformations and represent promising allosteric binding pockets based on their geometric and physicochemical properties as well as their druggability. The identified binding pocket acts as a new allosteric site and can be used for new drug development, alleviating the active pocket related problems encountered in the development of inhibitors for the NS2B/NS3 protease in *Flavivirus*.

## Figures and Tables

**Table 1:** Key structural features of the NS2B/NS3 protease model generated from AlphaFold.

Important Structural Properties			NS2B/NS3 ^a^	NS3 ^a^
MolProbity	atom contacts	clash score	**4.98 (94th percentile)**	**4.47 (95th percentile)**
	protein geometry	poor rotamers	**0 (0.00%)**	**0 (0.00%)**
		favored rotamers	144 (97.96%)	112 (97.39%)
		Ramachandran outliers	**0 (0.00%)**	**0 (0.00%)**
		Ramachandran favored	**182 (99.45%)**	**146 (99.32%)**
		MolProbity score	**1.26 (99th percentile)**	**1.22 (99th percentile)**
PROCHECK	Ramachandran plot	most favored	**138 (93.2%)**	**107 (91.5%)**
		additional allowed	**10 (6.8%)**	**10 (8.5%)**
		generously allowed	0 (0.0%)	0 (0.0%)
		disallowed	0 (0.0%)	0 (0.0%)

^a^ Bolded entries indicate more favorable characteristics.

**Table 2:** Contributions of the most important residues in NS2B/NS3 binding.

NS2B Residues	ΔG_bind_/kcal mol^−1^	NS3 Residues	ΔG_bind_/kcal mol^−1^
Trp12	−8.6	Arg44	−7.8
Leu2	−8.4	Tyr43	−4.6
Trp6	−7.3	Val80	−4.5
Val10	−5.5	Gln116	−4.4
Ala4	−4.9	Ile137	−4.3

**Table 3:** Cluster occupancy of NS2B/NS3 and NS3 conformations through time (1 μs).

System	Cluster	Conformation	Population	d ^a^	csd ^b^	RMSD against A
NS2B/NS3	1	A	0.636	2.394	0.285	0
	2	B	0.364	2.622	0.369	1.859
NS3	1	A	0.637	3.130	0.530	0
	2	B	0.363	3.226	0.670	3.537

^a^ d = average distance between points in the cluster, ^b^ csd = standard deviation of points in the cluster

**Table 4:** Binding pockets of conformations A and B of the NS2B/NS3 complex determined with the program DoGSiteScorer.

Conformation	Binding Pocket	Volume (Å^3^)	Surfaces (Å^2^)	Depth (Å)	Drug Score	Pocket Amino Acids
**A**	BP1	216.38	455.93	11.13	0.417	Thr3, Ala4 *, Glu5, Trp6 *, Tyr43 *, Lys64, Gly65, Val80*, Val81, Asp82, Glu83
	BP2	136	324.37	7.47	0.238	Gly20, Gly21, Glu22, Val23, Leu25, Pro148, Thr177, Glu179, Ala180, Val182
	BP3	134.34	310.16	7.87	0.235	Gly65, Val66, Leu79, Val81, Asp82, Ala84, Ile85, Ser86, Gly87, Tyr89, Tyr100, Gly101
	BP4	103.62	315.94	6.84	0.156	Glu22, Trp110, Gly112, Glu113, Thr114, Gln132, Pro133, Gly134, Glu135
**B**	BP1	320.19	533.63	14.34	0.620	Ala4 *, Glu5, Trp6 *, Lyn39, Tyr43 *, Gly62, Ala63, Lyn64, Gly65, Val66, Leu67, Leu79, Val81, Asp82, Ser86, Tyr100, Gly101
	BP2	270.14	530.71	14.72	0.595	Val66, Leu67, Hie68, Cys99, Tyr100, Gly102, Ala103, Trp104, Ser105, Leu106, Glu107, Ser108, Arg109, Gln187
	BP3	259.14	484.09	11.22	0.450	Glu9, Val10, Glu11, Asp40, Gly41, Tyr61, Gly62, Ala63, Trp104, Ser105, Leu106, Glu107, Gly165, Glu166, Val167
	BP4	179.71	392.03	9.69	0.309	Val27, Arg28, Gln29, Asp30, Ala31, Gly33, Arg94, Glu95, Leu138, Glu139, Asn140, Arg142
	BP5	122.3	254.17	6.34	0.183	Gly20, Gly21, Glu22, Val23, Pro148, Thr177, Glu179, Ala180, Val182

* Key residues that play important roles in NS2B/NS3 binding based on MM/GBSA analysis

**Table 5:** Non-covalent docking binging scores (in kcal mol^−1^) of 4 hit compounds in the binding pockets (BP1) of conformations A of the KFDV NS2B/NS3 complex.

Compound Name	Structural Formula	Drug Bank ID	A	B	Score
zosuquidar	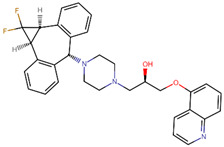	DB06191	−8.4	−6.7	−7.8
pramiconazole	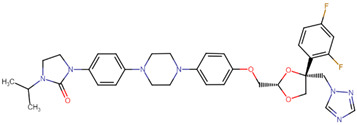	DB06295	−8.3	−7.8	−8.1
2-(4-fluorophenyl)-*N*-{[3-fluoro-4-(1*H*-pyrrolo [2,3-*b*]pyridin-4-yloxy)phenyl]carbamoyl}acetamide	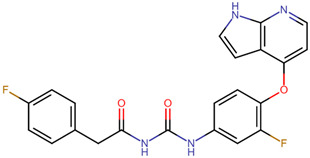	DB06997	−8.4	−8.1	−8.3
MK-3207	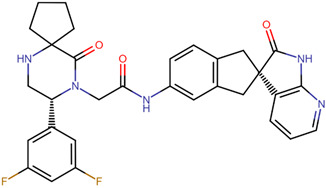	DB12424	−8.8	−8.4	−8.7

**Table 6:** List of amino acids with a contribution below −1.0 kcal mol^−1^ to the binding free energy (ΔG_bind_) for the binding of DB06191, DB06295, DB06997, and DB12424 to the NS2B/NS3 protease. Energies are expressed in kcal mol^−1^ as mean ± standard deviation.

DB06191		DB06295		DB06997		DB12424	
Residue	ΔG_bind_	Residue	ΔG_bind_	Residue	ΔG_bind_	Residue	ΔG_bind_
Leu50	−1.87 ± 0.55	Trp6	−2.56 ± 0.22	Trp6	−2.32 ± 0.09	Trp6	−2.89 ± 0.10
His54	−1.32 ± 0.42	Val81	−2.56 ± 0.22	Thr3	−1.42 ± 0.10	Glu5	−1.64 ± 0.08
Pro48	−1.28 ± 0.14	Asp82	−1.83 ± 0.22			Val81	−1.35 ± 0.22
		Lyn64	−1.55 ± 0.22			Thr3	−1.20 ± 0.02
		Val80	−1.49 ± 0.46				
		Glu83	−1.40 ± 0.13				

## Data Availability

The computational data presented in this study are openly available in BIOTECHRI repository at https://urn.nsk.hr/urn:nbn:hr:193:208603 (accessed on 24 May 2023).

## Supplementary Materials

The supporting information can be downloaded at: https://www.mdpi.com/article/10.3390/ijms241310907/s1. References [45,46,47,57,58,71,72,73,74,75] are cited in the supplementary materials.
