# Supplementary material for: Structural Modifications Introduced by NS2B Cofactor Binding to the NS3 Protease of the Kyasanur Forest Disease Virus
Source: Int J Mol Sci. 2023 Jun 30;24(13):10907. doi: 10.3390/ijms241310907 (PMC10342073; doi:10.3390/ijms241310907)
Supplement: Supplementary file 1 [file ijms-24-10907-s001.zip › Supplementary Information.pdf]

International Journal of *Molecular Sciences*

Article

Structural Modifications Introduced by NS2B Cofactor Binding to the NS3 Protease of the Kyasanur Forest Disease Virus

Shivananda Kandagalla [1,*,†], Bhimanagoud Kumbar [2] and Jurica Novak [3,4,*,†]

[1] Laboratory of Computational Modeling of Drugs, Higher Medical & Biological School, South Ural State University, 454080 Chelyabinsk, Russia
[2] ICAR-National Institute of Veterinary Epidemiology and Disease Informatics, Bengaluru 560064, Karnataka, India; kumbar.bhimanagoud@gmail.com
[3] Department of Biotechnology, University of Rijeka, 51000 Rijeka, Croatia
[4] Center for Artificial Intelligence and Cybersecurity, University of Rijeka, 51000 Rijeka, Croatia
* Correspondence: kandagallas@susu.ru or kandagallas@gmail.com (S.K.); jurica.novak@uniri.hr (J.N.)
† These authors contributed equally to this work.

Supplementary Figures and Tables:

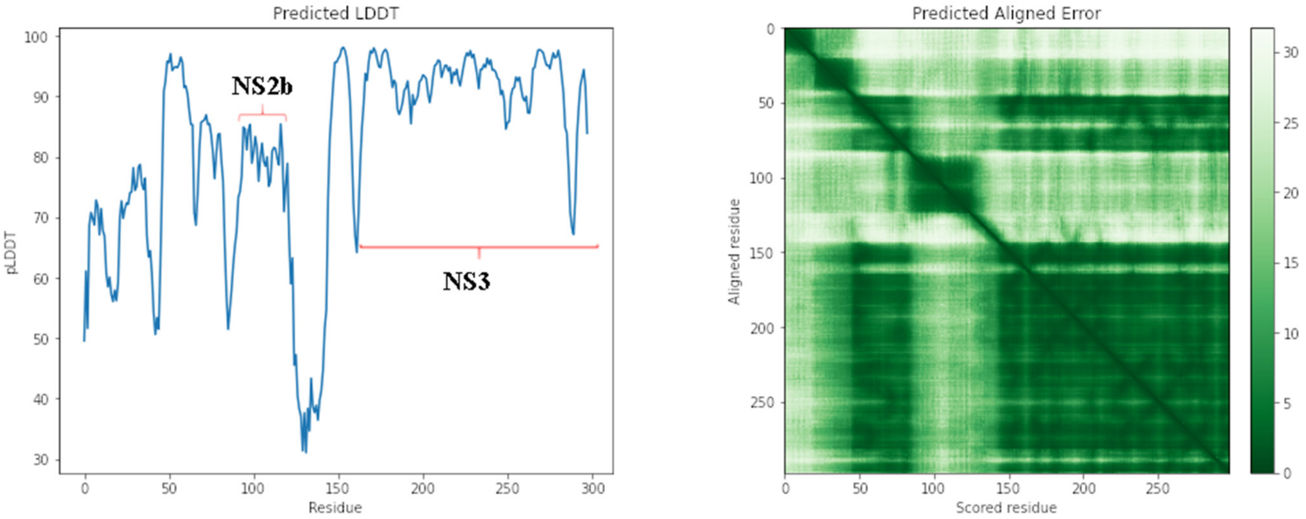

Figure S1. Confidence score of the NS2B/NS3 protease model obtained by AlphaFold.

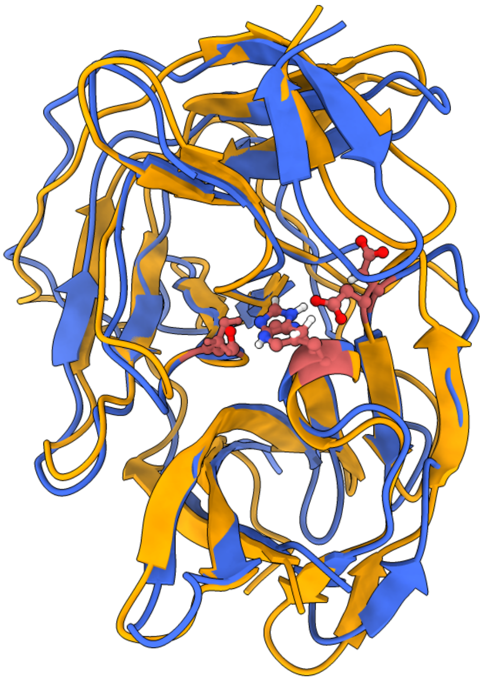

Figure S2. Superimposed structures of Zika NS3 protease (6PJW, orange) and KFDV NS3 (blue). The substrate binding site (or catalytic triad) is highlighted in red.

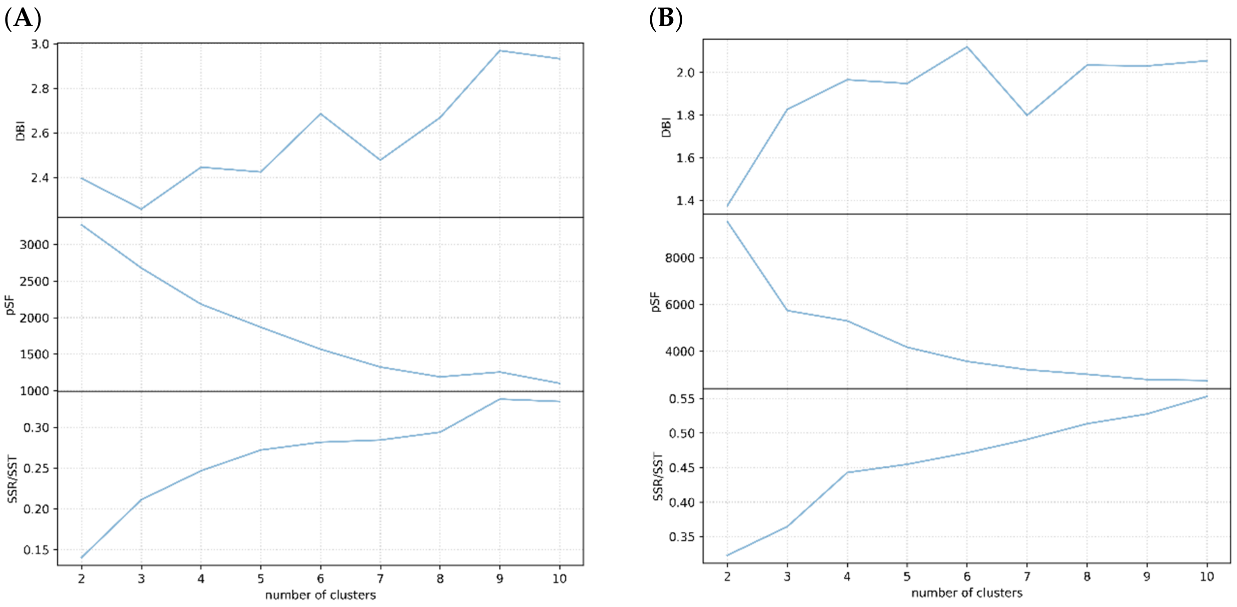

Figure S3. Graphs showing the values of the clustering metrics DBI (The Davies-Bouldin index), pSF (the pseudo-F statistic), and SSR/SST (the ratio of sum of squares regression and sum of squares error) for the MD trajectory of NS2B/NS3 complex (**A**) and NS3 (**B**).

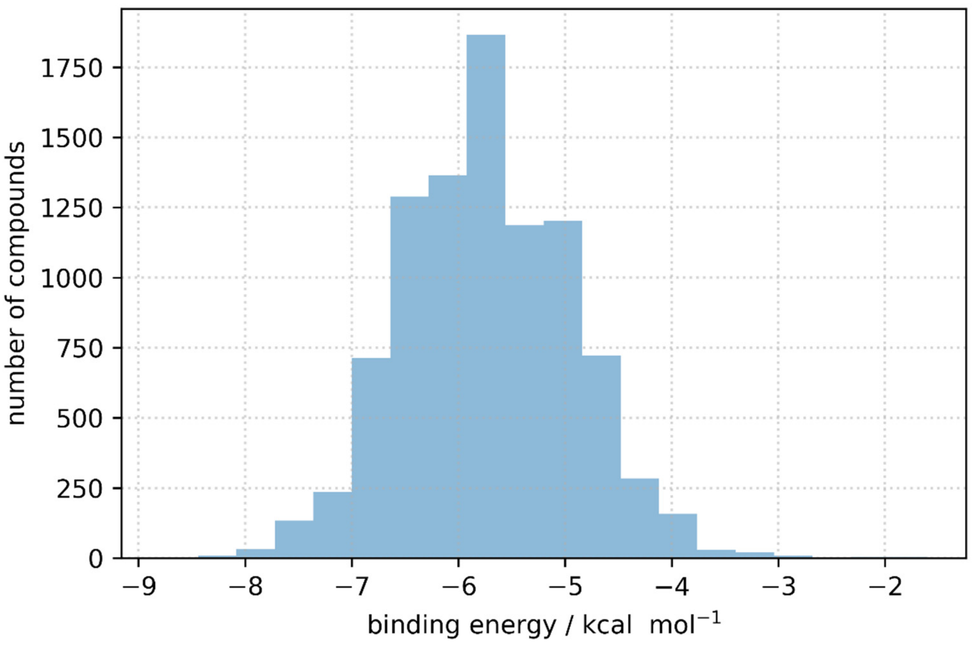

Figure S4. Distribution of binding energy of the potential allosteric inhibitors against the binding pocket (BP1) NS2B/NS3 conformation A.

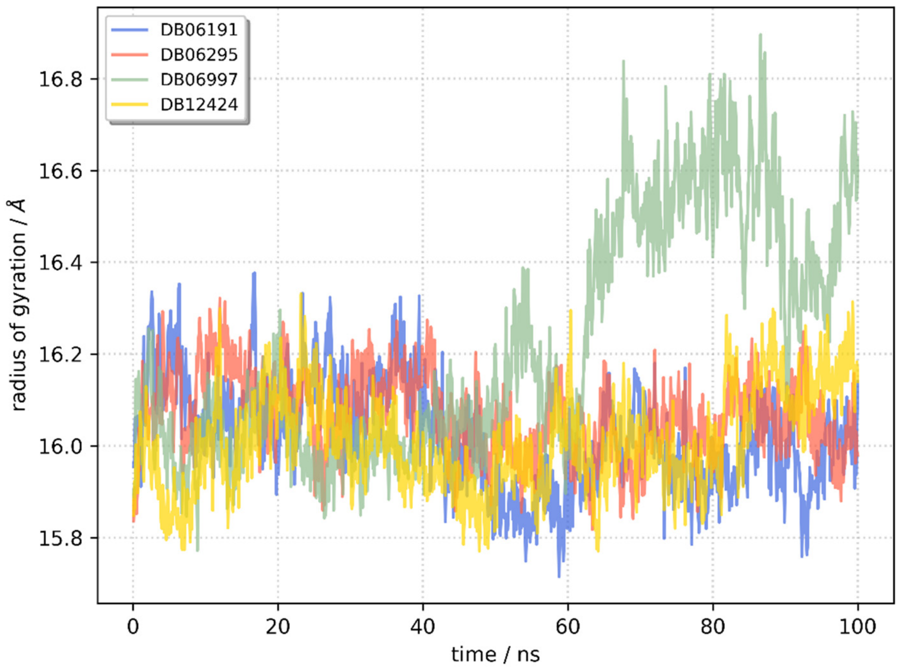

Figure S5. Radius of gyration analysis of the MD trajectories of KFDV NS2B/NS3 protease with potential allosteric inhibitors.

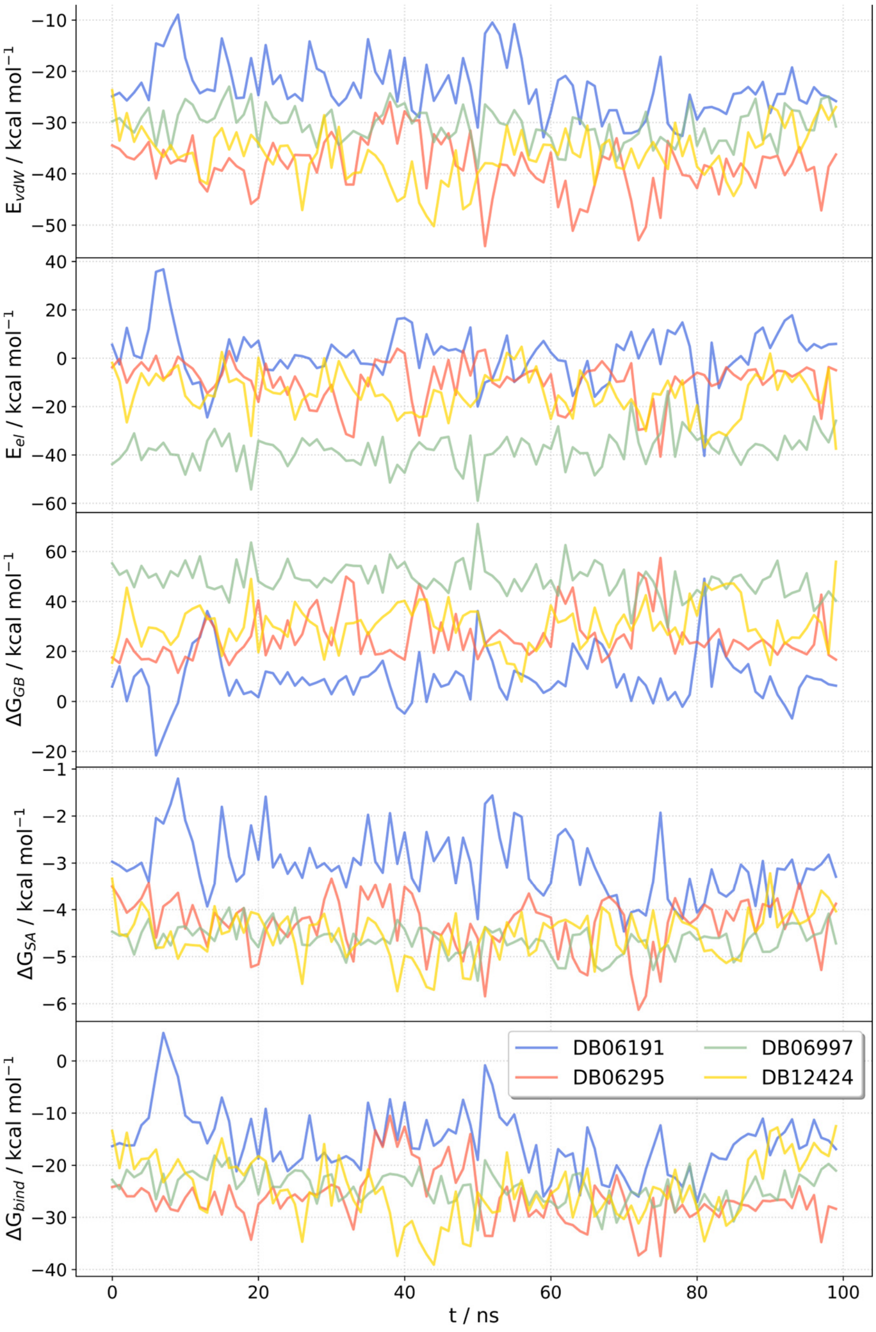

Figure S6. The energy contributions to the free energy of binding were analyzed for the top four compounds when bound to the allosteric binding pocket of NS2B/NS3 of KFDV.

Table S1. Energy analysis for binding of hit compounds to allosteric site of NS2B/NS3 of as obtained by MM/PBSA method. All units are kcal mol^{-1}.

Compound	ΔG_{bind}	ΔE_{vdW}	$\Delta E_{electrostatic}$	ΔG_{GB}	ΔG_{SA}
DB06191	−15.5 ± 5.9	−23.1 ± 5.4	1.6 ± 10.9	9.1 ± 10.0	−3.0 ± 0.7
DB06295	−26.3 ± 4.9	−38.5 ± 5.2	−9.5 ± 8.7	26.0 ± 9.4	−4.3 ± 0.6
DB06997	−24.2 ± 3.1	−30.6 ± 3.5	−37.7 ± 6.7	48.7 ± 6.3	−4.6 ± 0.3
DB12424	−24.6 ± 6.0	−36.2 ± 5.1	−14.6 ± 9.0	30.8 ± 8.6	−4.5 ± 0.5

ΔG_{bind}—free energy of binding (without entropic contribution), van der Waals (ΔE_{vdW}) and electrostatic ($\Delta E_{electrostatic}$) energy, polar electrostatic solvation energy (ΔG_{GB}), nonpolar, nonelectrostatic solvation energy (ΔG_{SA}).

Table S2. Noncompetitive inhibitors of flavivirus NS2B/NS3 protease.

Compound	Targeted Virus*	Method Used to for Identification Allosteric Binding Pocket	PDB ID Used for Modeling	Reference
temoporfin	ZIKV<br>DENV-2	induced fit docking (IFD) protocol	ZIKV (PDB ID: 5LC0)<br>DENV3 (PDB ID: 3U1I)	[1]
niclosamide	ZIKV<br>DENV-2	molecular docking	ZIKV (PDB ID: 5LC0)<br>DENV3 (PDB ID: 3U1I)	[1]
nitazoxanide	ZIKV<br>DENV-2	molecular docking	ZIKV (PDB ID: 5LC0)<br>DENV3 (PDB ID: 3U1I)	[1]
methylene blue	DENV-2<br>ZIKV	-	-	[2]
erythrosin B	ZIKV<br>DENV-2<br>WNV<br>JEV<br>YFV	induced fit docking (IFD) protocol	ZIKV (PDB ID: 5LC0)<br>DENV3 (PDB ID: 3U1I)	[3]
JMX0902	Zika	-	-	[4]
JMX0207	ZIKV<br>DENV	induced fit docking (IFD) protocol	DENV2<br>(PDB Code: 2FOM)	[5]
NSC135618	DENV-2<br>ZIKV<br>WNV<br>YFV		DENV2<br>(PDB Code: 2FOM)	[6]
(4-(3-(4-(furan-3-yl)phenyl)-5-(piperidin-4-ylmethoxy)pyrazin-2-yl)phenyl)methanamine hydrochloride	ZIKV<br>DENV-2<br>DENV-3<br>WNV	X-ray crystallography	-	[7,8]
(4-(5-(piperidin-4-ylmethoxy)-3-(4-(tetrahydro-2H-pyran-4-yl)-phenyl)pyrazin-2-yl)phenyl)methanamine hydrochloride	ZIKV<br>DENV-2<br>WNV	-	-	[8]
2-(2-chlorophenyl)benzo[d]isothiazol-3(2*H*)-one	DENV-2	molecular docking	DENV3 NS2NS3<br>(PDB ID: 3U1I)	[9]
2-(2-methyl-4-nitrophenyl)benzo[d]isothiazol-3(2*H*)-one	DENV-2	molecular docking	DENV3 NS2NS3<br>(PDB ID: 3U1I)	[9]
2-(2,6-dichlorophenyl)benzo[d]isothiazol-3(2*H*)-one	DENV-2	molecular docking	DENV3 NS2NS3<br>(PDB ID: 3U1I)	[9]
(*R*)-*N*-(5,6-dihydroxybenzo[d]thiazol-2-yl)-1-((4-nitrophenyl)- sulfonyl)pyrrolidine-2-carboxamide	DENV-2	molecular docking followed by molecular dynamic simulation	DENV3 NS2NS3<br>(PDB ID:2FOM)	[10]
(*S*)-*N*-(5,6-dihydroxybenzo[d]-thiazol-2-yl)-1-tosylpiperidine-2- carboxamide	DENV-2	molecular docking followed by molecular dynamic simulation	DENV3 NS2NS3 (PDB ID:2FOM)	[10]
(*R*)-*N*-(5,6-dihydroxybenzo[d]-thiazol-2-yl)-1-tosylpiperidine-2- carboxamide	DENV-2	molecular docking followed by molecular dynamic simulation	DENV3 NS2NS3 (PDB ID:2FOM)	[10]

* DENV-2 = dengue virus serotype-2, DENV-3 = dengue virus serotype-3, WNV = West Nile virus, YFV = yellow fever virus, ZIKV = Zika virus.

References

1. Li, Z.; Brecher, M.; Deng, Y.Q.; Zhang, J.; Sakamuru, S.; Liu, B.; Huang, R.; Koetzner, C.A.; Allen, C.A.; Jones, S.A.; et al. Existing Drugs as Broad-Spectrum and Potent Inhibitors for Zika Virus by Targeting NS2B-NS3 Interaction. *Cell Research* **2017**, *27*, 1046–1064, doi:10.1038/cr.2017.88.
2. Li, Z.; Lang, Y.; Sakamuru, S.; Samrat, S.; Trudeau, N.; Kuo, L.; Rugenstein, N.; Tharappel, A.; D'Brant, L.; Koetzner, C.A.; et al. Methylene Blue Is a Potent and Broad-Spectrum Inhibitor against Zika Virus in Vitro and in Vivo. *Emerging Microbes & Infections* **2020**, *9*, 2404, doi:10.1080/22221751.2020.1838954.
3. Li, Z.; Sakamuru, S.; Huang, R.; Brecher, M.; Koetzner, C.A.; Zhang, J.; Chen, H.; Qin, C. feng; Zhang, Q.Y.; Zhou, J.; et al. Erythrosin B Is a Potent and Broad-Spectrum Orthosteric Inhibitor of the Flavivirus NS2B-NS3 Protease. *Antiviral research* **2018**, *150*, 217, doi:10.1016/J.ANTIVIRAL.2017.12.018.
4. Li, Z.; Xu, J.; Lang, Y.; Wu, X.; Hu, S.; Samrat, S.K.; Tharappel, A.M.; Kuo, L.; Butler, D.; Song, Y.; et al. In Vitro and in Vivo Characterization of Erythrosin B and Derivatives against Zika Virus. *Acta Pharmaceutica Sinica B* **2022**, *12*, 1662–1670, doi:10.1016/J.APSB.2021.10.017.
5. Li, Z.; Xu, J.; Lang, Y.; Fan, X.; Kuo, L.; D'Brant, L.; Hu, S.; Samrat, S.K.; Trudeau, N.; Tharappel, A.M.; et al. JMX0207, a Niclosamide Derivative with Improved Pharmacokinetics, Suppresses Zika Virus Infection Both in Vitro and in Vivo. *ACS Infectious Diseases* **2020**, *6*, 2616–2628, doi:10.1021/acsinfecdis.0C00217.
6. Brecher, M.; Li, Z.; Liu, B.; Zhang, J.; Koetzner, C.A.; Alifarag, A.; Jones, S.A.; Lin, Q.; Kramer, L.D.; Li, H. A Conformational Switch High-Throughput Screening Assay and Allosteric Inhibition of the Flavivirus NS2B-NS3 Protease. *PLOS Pathogens* **2017**, *13*, e1006411, doi:10.1371/JOURNAL.PPAT.1006411.
7. Yao, Y.; Huo, T.; Lin, Y.L.; Nie, S.; Wu, F.; Hua, Y.; Wu, J.; Kneubehl, A.R.; Vogt, M.B.; Rico-Hesse, R.; et al. Discovery, X-Ray Crystallography and Antiviral Activity of Allosteric Inhibitors of Flavivirus NS2B-NS3 Protease. *Journal of the American Chemical Society* **2019**, *141*, 6832–6836, doi:10.1021/JACS.9B02505.
8. Nie, S.; Yao, Y.; Wu, F.; Wu, X.; Zhao, J.; Hua, Y.; Wu, J.; Huo, T.; Lin, Y.L.; Kneubehl, A.R.; et al. Synthesis, Structure-Activity Relationships, and Antiviral Activity of Allosteric Inhibitors of Flavivirus NS2B-NS3 Protease. *Journal of Medicinal Chemistry* **2021**, *64*, 2777–2800, doi:10.1021/ACS.JMEDCHEM.0C02070.
9. Batool, F.; Saeed, M.; Saleem, H.N.; Kirschner, L.; Bodem, J. Facile Synthesis and in Vitro Activity of N-Substituted 1,2-Benzisothiazol-3(2H)-Ones against Dengue Virus NS2BNS3 Protease. *Pathogens* **2021**, *10*, 464, doi:10.3390/PATHOGENS10040464.
10. Millies, B.; Von Hammerstein, F.; Gellert, A.; Hammerschmidt, S.; Barthels, F.; Göppel, U.; Immerheiser, M.; Elgner, F.; Jung, N.; Basic, M.; et al. Proline-Based Allosteric Inhibitors of Zika and Dengue Virus NS2B/NS3 Proteases. *Journal of Medicinal Chemistry* **2019**, *62*, 11359–11382, doi:10.1021/ACS.JMEDCHEM.9B01697.
